# Supplementary material for: Structural–Functional Relationship of the Ribonucleolytic Activity of aIF5A from Sulfolobus solfataricus
Source: Biomolecules. 2022 Oct 6;12(10):1432. doi: 10.3390/biom12101432 (PMC9599864; doi:10.3390/biom12101432)
Supplement: Supplementary file 1 [file biomolecules-12-01432-s001.zip › biomolecules-1899193-supplementary.pdf]

Structural-Functional Relationship of the Ribonucleolytic Activity of aIF5A from *Sulfolobus solfataricus*

Alice Romagnoli [1,2,*], Paolo Moretti [1], Mattia D'Agostino [1], Jesmina Rexha [1], Nunzio Perta [1], Astra Piccinini [1], Daniele Di Marino [1,2], Francesco Spinozzi [1], Anna La Teana [1,2,*]

[1] Department of Life and Environmental Sciences, Polytechnic University of Marche, Via Brecce Bianche, 60131 Ancona, Italy
[2] New York-Marche Structural Biology Center (Ny-MaSBiC), Polytechnic University of Marche, Via Brecce Bianche, 60131, Ancona, Italy.
* Corresponding:a.lateana@univpm.it or a.romagnoli@univpm.it

Supplementary materials

SAXS data analysis

Guinier/Porod analysis

All experimental SAXS curves for both recombinant N-His-aIF5A samples alone (first series) and in the presence of rRNA (second series) at low q show a marked positive curvature trend, indicating the presence of large aggregates, in combination with a mild negative curvature behavior, representative of smaller particles. Accordingly, all the curves have been firstly analyzed by a linear combination of Guinier and Porod asymptotic behaviors,

$$\frac{d\Sigma}{d\Omega}(q) = K_G e^{-\frac{q^2 R_g^2}{3}} + K_P q^{-4}, \qquad \text{(S1)}$$

where R_g is the radius of gyration of smaller particles. Notice that the validity of this approximation holds for $q_{\max} R_g \leq 1.3$, as widely known from scattering theory [1]. For samples only containing protein (first series of SAXS curves), the Guinier constant K_G allows to calculate the apparent aggregation number, $N_{agg,sml}$, of smaller particles, according to

$$K_G = \frac{\mathring{c}_{5A} N_A d_{wat}}{M_{5A}} \left(\frac{v_{5A}}{\phi_{5A}}\right)^2 \left(\rho_{5A}\phi_{5A} - \rho_{solv}\big(1 - d_h(1-\phi_{5A})\big)\right)^2 N_{agg,sml} \qquad \text{(S2)}$$

In this equation N_A is Avogadro's number, $\mathring{c}_{5A}$ is the w/v concentration of 5A at the reference temperature T_0=25°C and M_{5A} is the molecular weight of the monomer 5A. The term d_{wat} represents the relative mass density of water with respect its value at T_0here calculated according to

$$d_{wat} = e^{-\alpha_{wat}(T-T_0) - \beta_{wat}(T-T_0)^2/2} \qquad \text{(S3)}$$

where the best values of the thermal expansivity of water and its first derivative, both at T_0, are $\alpha_{wat} = 2.5 \cdot 10^{-4}$ K^{-1} and $\beta_{wat} = 9.8 \cdot 10^{-6}$ K^{-2}, respectively [2].

The term v_{5A} is the molecular volume of dry 5A, which depends on temperature according to

$$v_{5A} = v^\circ{}_{5A}\, e^{\alpha_{5A}(T-T_0)} \qquad \text{(S4)}$$

where $\alpha_{5A} = 1.15 \cdot 10^{-4}$ K^{-1} is the dry protein thermal expansivity factor [3]. The volume fraction of dry protein with respect to the volume of hydrated protein is here represented by ϕ_{5A}. For globular proteins, this fraction is in the order of 0.6 [4].

The scattering length densities (SLDs) of protein and solvent are written as a function of temperature and solvent composition, expressed in terms of molar concentration of KCl ($C^\circ{}_{KCl}$) and volume fraction of glycerol ($c_V{}^\circ{}_{gly}$) at the reference temperature, according to

$$\rho_{5A} = r_e \frac{e_{5A}}{v_{5A}} \qquad \text{(S5)}$$

$$\rho_{solv} = \frac{d_{wat}}{d^\circ_{wat}} r_e \left(\left(1 - N_A C^\circ_{KCl} (v^\circ_{K^+} + v^\circ_{Cl^-}) - c_{\mathrm{V}}{}^\circ_{gly}\right) \frac{e_{wat}}{v^\circ_{wat}} + c_{\mathrm{V}}{}^\circ_{gly} \frac{e_{gly}}{v^\circ_{gly}} + N_A C^\circ_{KCl} (e_{K^+} + e_{Cl^-}) \right) \quad \text{(S6)}$$

where $r_e = 0.28 \cdot 10^{-12}$ cm is the classical radius of the electron, e_j represents the number of electrons of the j-molecule and v_j its molecular volume. Equation S2 has been written considering the water hydration around the proteins, which, in general, shows a relative mass density (here indicated by d_h) higher than the one of bulk water.

QUAFIT

The unknown structure of N-His-aIF5A from *E. coli* monomer in solution has been estimated by using the QUAFIT method [5] considering that the protein is constituted by two rigid domains (the N-terminal domain, containing the strands b1-b6, and the C-terminal domain, from b7 to b11 strand) linked by a hinge linker (between b1 and b7) and also including a flexible fragment formed by the 6 His residues. QUAFIT, in general, realizes a SAXS curves fitting, optimizing the relative position of the rigid domains and the angles of Ramachandran relative to the flexible domains. It is possible to analyze the SAXS curve assuming that the protein can be distributed over multiple structures, each having a distinct relative position of the rigid domains and distinct conformations of the flexible domains. Unfortunately, the presence of aggregated form of N-His-aIF5A, that mostly affects SAXS curves at low q, does not allow a robust determination of the most suitable conformations representing the N-His-aIF5A monomer. Hence, we used the QUAFIT method only as a bioinformatics tool to select, in a semi-quantitative way, 10 representative conformations of the monomer N-His-aIF5A, adopting, as a constraint, the high q region of the experimental SAXS curve at at 10 mg/mL protein, T=63°C, 60 mM KCl. The 10 conformations are shown in Figure S4: in red is represented the N-terminal domain, in green the C-terminal domain, while in blue the extra amino acids of the protein (6 x His-tag, TEV site). To note, QUAFIT allows to calculate the SAXS form factor $P_j(q)$ of each of them.

GENFIT

The macroscopic differential scattering cross section (SCS) of a system constituted by large protein aggregates and interacting protein monomers distributed over N conformations, is

$$\frac{d\Sigma}{d\Omega}(q) = \frac{c^\circ_{5A} N_A d_{wat}}{M_{5A}} [x_{por} \frac{d\Sigma}{d\Omega_{\mathrm{por}}}(q) + (1 - x_{por}) S_M(q) \sum_{j=1}^{N} w_j P_j(q)] \quad \text{(S7)}$$

In this equation, x_{por} represents the fraction of all the 5A monomers aggregated in large particles. Their SCS, in absolute scale, is written by considering all the factors of the Porod law [6],

$$\frac{d\Sigma}{d\Omega_{\mathrm{por}}}(q) = v_{5A} (\rho_{5A} - \rho_{solv})^2 \frac{2\pi\sigma}{q^4} \quad \text{(S8)}$$

$$\sigma = 4\pi \frac{\left(\left(3(4\pi)^{-1} N_{agg,por} v_{5A} \right)^{1/3} \right)^2}{N_{agg,por}\, v_{5A}} \quad \text{(S9)}$$

where σ represents the surface/volume ratio of the aggregate, here calculated considering the geometry of a sphere, and $N_{agg,por}$ is the Porod's particle aggregation number. It is expected that $N_{agg,por}$ changes with all the samples compositions parameters, namely with c°_{5A}, C°_{kcl} and temperature. In a simple approximation, here we assume that its logarithm is a linear function of all these parameters,

$$\log N_{agg,por} = N^\circ_{agg,por} \left(1 + \xi^\circ_{\mathrm{N,c_{5A}}} (c^\circ_{5A} - c^{\mathrm{min}}_{5A}) + \xi^\circ_{N,C_{KCl}} (C^\circ_{KCl} - C^{\mathrm{min}}_{KCl}) + \xi^\circ_{N,T} (T - T^{\mathrm{min}}) \right) \quad \text{(S10)}$$

Moreover, for the sake of simplicity, we have not considered any effect of the hydration shell around these large aggregates.

Considering the N conformations of the protein monomers selected by QUAFIT, their relative weights w_j are calculated by a classical thermodynamic scheme,

$$w_j = \frac{K_{1,j}}{D} \quad \text{(S11)}$$

$$D = \sum_{j=1}^{N} K_{1,j} \quad \text{(S12)}$$

where $K_{1,j}$ represents the equilibrium constant of the transformation of the 5A monomer from the conformation 1 to the conformation j. The dependency of $K_{1,j}$ on temperature and KCl concentration is here written according to the following expressions,

$$K_{1,j} = \exp\left(-\frac{\Delta G°_{j,1}}{\mathrm{R}T}\right) \quad \text{(S13)}$$

$$\Delta G°_{j,1} = \Delta H°_{j,1}\left(1 + \xi°_{H,j}\left(C°_{KCl} - C_{KCl}^{\min}\right)\right) - T\Delta \mathrm{S}°_{\mathrm{j,1}}\left(1 + \xi°_{S,j}\left(C°_{KCl} - C_{KCl}^{\min}\right)\right) \quad \text{(S14)}$$

where $\xi_{H,j}$ and $\xi_{S,j}$ represent the first derivative with respect to $C°_{KCl}$ of the enthalpy and the entropy changes, respectively, at T_0, both divided by their respective values, $\Delta H°_{j,1}$ and $\Delta S°_{j,1}$, at T_0 and at the minimum value $C_{KCl}^{\min}$ of KCl. R is the ideal gas constant.

The function $S_M(q)$ in equation 8 is the effective (also called ``measured'') structure factor, representing the average interactions among the monomers at any conformation. According to [2], it is approximated by the following equations

$$S_M(q) = 1 + \beta(q)[S(q) - 1] \quad \text{(S15)}$$

$$\beta(q) \approx \sum_{j=1}^{N} w_j \frac{\left|P_j^1(q)\right|^2}{P_j(q)} \quad \text{(S16)}$$

where $P_j^1(q)$ is the orientational average of the scattering amplitude of the j-conformer. We have considered a unique monomer-monomer structure factor $S(q)$, which is the Fourier transform of the average monomer-monomer correlation function $g(r)$. The $S(q)$ is calculated on the basis of the pair potential $u(r)$, written as a combination of a hard-sphere potential and two Yukawian potentials (HSDY), one representing the screened electrostatic repulsion and the other one the van der Waals attraction. The calculation of $S(q)$ is performed by applying the Random Phase Approximation (RSA) of the Mean Spherical Approximation (MSA). All details are reported, for example, in [2].

The parameters of the protein-protein interaction that enter in the calculation of $S(q)$ are the effective monomer radius R, the number of elementary charges, Z, of the 5A, the ionic strength of the solution, I_S, the relative dielectric constant of the solution, here approximated by the dielectric constant of water, whose dependency on temperature is known [7], the depth J of the attractive potential at contact ($r = 2R$) and the range d of its exponential decay.

The 5A radius is taken as the radius of a spheres of volume v_{5A}, corrected by a factor γ that can assume values close to 1, $R = \gamma\left(\frac{3v_{5A}}{4\pi}\right)^{1/3}$. The protein charge Z can be calculated on the basis of the solution pH, by considering the primary sequence of 5A and by taking into account the known values of acidic constants pK_a of the 20 amino-acids. Also I_S is easily calculated on the basis of sample compositions. On the contrary, J and d are difficult to estimate: their values are obtained by the fitting of SAXS data, considering, for the sake of simplicity, a linear variation with $C°_{KCl}$ and temperature.

The SAXS data fitting according to Eq. S7 and the set of all the others equations reported above has been carried out by means of the GENFIT software.

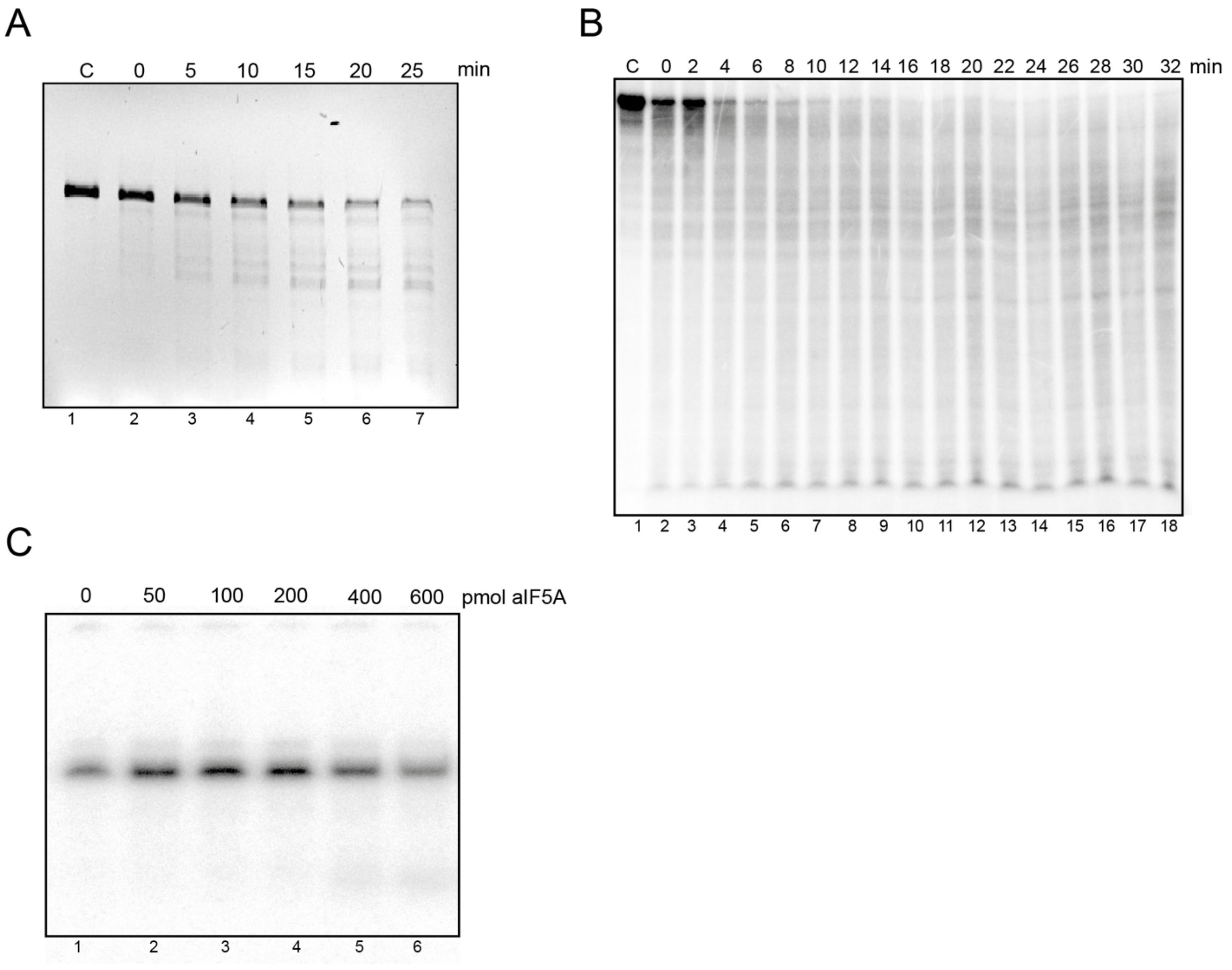

Figure S1. RNA degradation assays. (A) Degradation of *S. solfataricus* 0910 mRNA (time course from 0 to 25 minutes, lane 2-7) after incubation with recombinant N-His-aIF5A (produced in *E. coli*) at 65°C, lane 1: RNA incubated for 25 min at 65°C in absence of proteins. After incubation, samples were loaded on 8% PAA 8M Urea gels; (B) Radioactive assay for the degradation of 5′-labelled 0910 mRNA after aIF5A incubation performed as in [8]. Lane 1: incubation of 5′-[γ-32P] ATP-0910 mRNA without protein for 32 min at 65°C. Lanes 2-18: time course of degradation of 5′-[γ-32P] ATP -0910 mRNA at 65°C in the presence of *S. solfataricus* N-His-aIF5A purified from *E. coli.* Every 2 minutes, an aliquot was withdrawn and loaded on 8% PAA 8M Urea gels. (C) 5 pmol of 5′-[γ-32P] ATP-ncRNA98 were incubated with different amount of *S. solfataricus* N-His-aIF5A, from 0 (lane 1) to 400 pmol (lane 6) for 25 min at 65°C and loaded on a 8% PAA gel. In (B) and (C) RNAs were radioactively labelled at the 5′ end as previously performed [8].

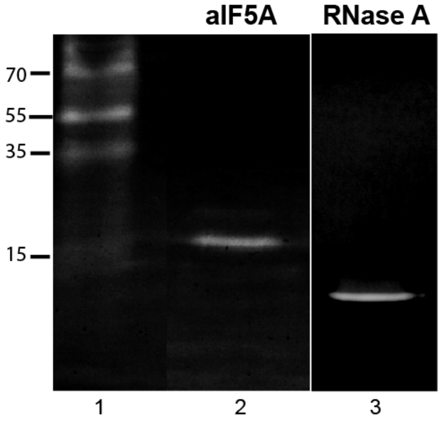

Figure S2. Zymogram assay for the identification of RNase activity of aIF5A. A mixture of rRNA in DEPC water was added into the gel matrix at a final concentration of 0.15 mg/mL. Lane 1: molecular weight standard; lane 2: 3 µg of *S. solfataricus* aIF5A [9] lane 3: 1 ng of bovine pancreatic RNase A (Merck Millipore), as a positive control, were loaded onto the gel and separated on a 15% SDS polyacrylamide gel. The assay was performed as in [8].

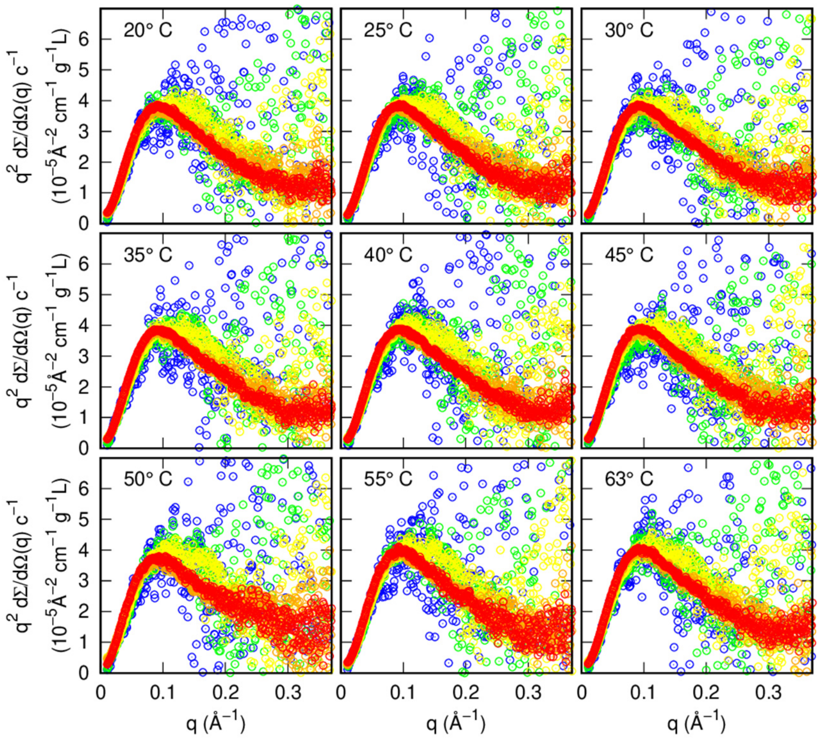

Figure S3. Kratky plots of SAXS curves of N-His-aIF5A with 1000 mM KCl. Curve have been divided by the protein concentration.

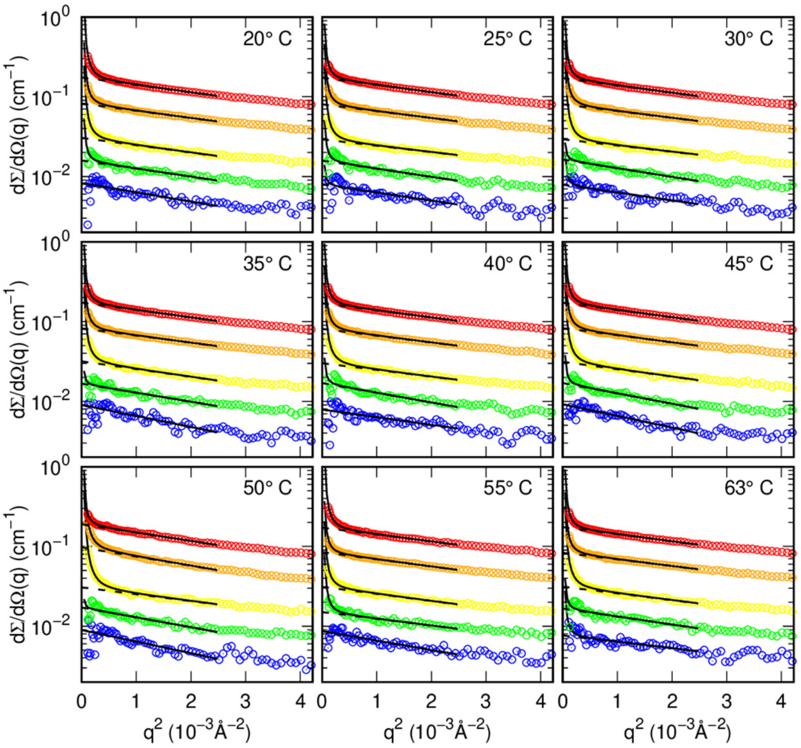

Figure S4. Guinier plots of SAXS curves of N-His-aIF5A with 1000 mM KCl. The solid black lines are the best Guinier/Porod fits (Eq. S1 in the Supporting Material), whereas the dashed black lines are the Guinier law contribution (first term of Eq. S1).

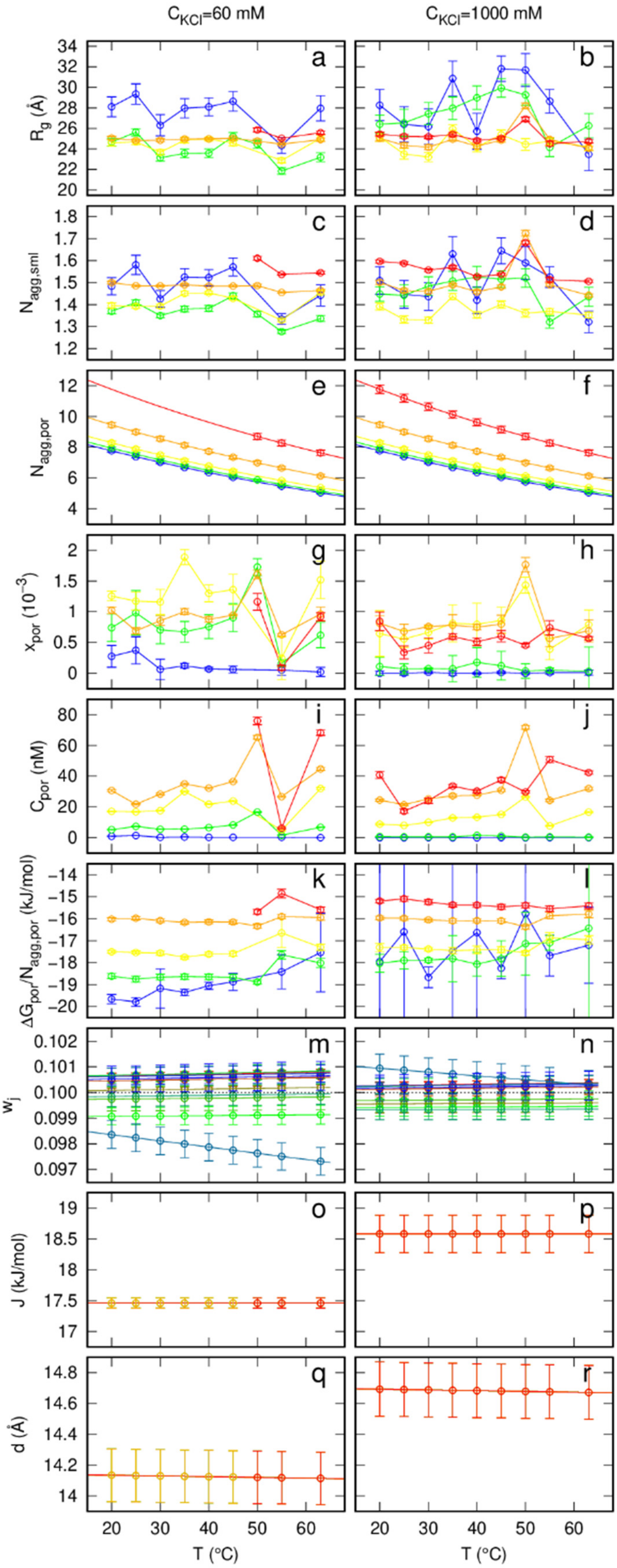

Figure S5. Temperature dependency of the fitting parameters obtained with the Guinier/Porod analysis (panels a-d) and with the GENFIT analysis (panels e-r) of the SAXS curve of N-His-aIF5A. The first and the second column of frames refer to samples with 60 and 1000 mM KCl, respectively, as indicated.

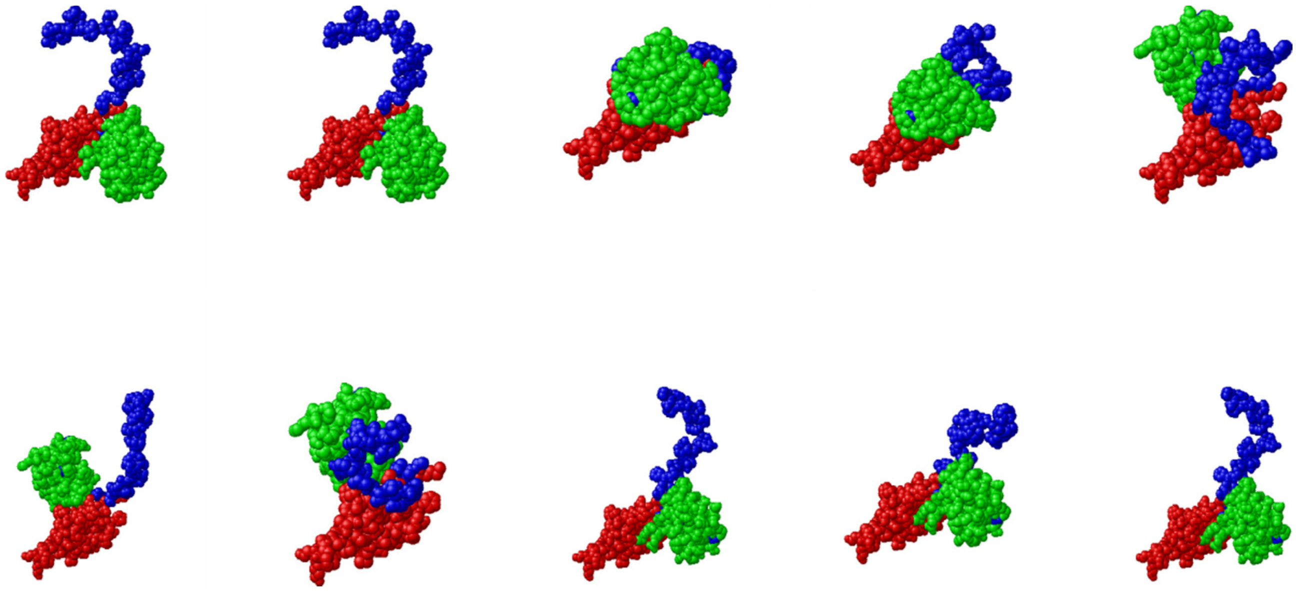

Figure S6. Spacefill representation of the 10 conformers of N-His-aIF5A selected by QUAFIT. The following domains was defined, through homology sequence with analogous proteins [10]: first rigid domain: residues 27-93 (N-terminal domain, red); second rigid domain: residues 98-154 (C-terminal domain, green); first flexible linker: residues 1-26 (His tag, blue); second flexible linker: residues 94-97 (blue); third flexible linker: residues 155-157 (blue).

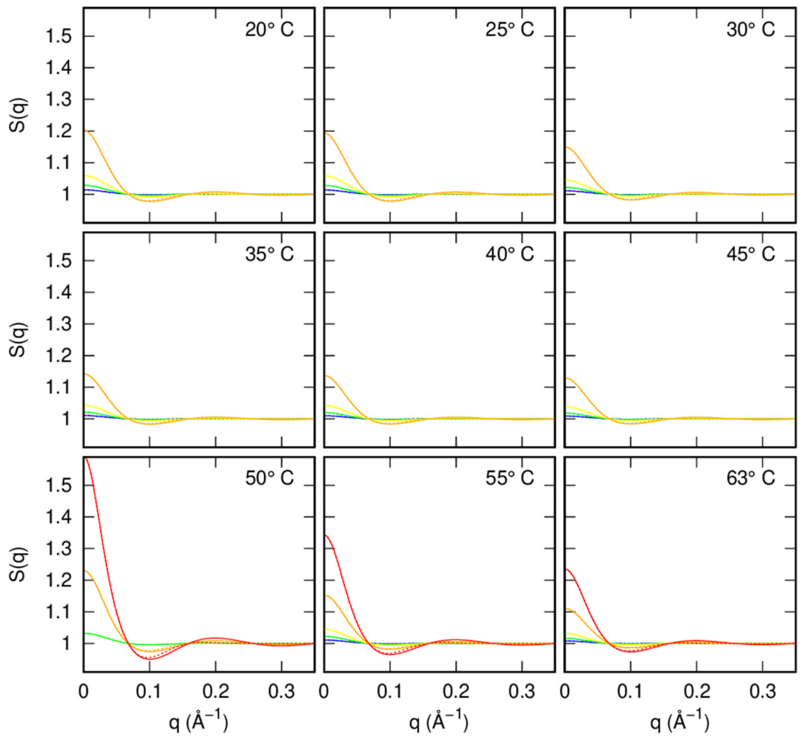

Figure S7. Structure factors $S(q)$ (solid lines) and "measured" structure factors $S_M(q)$ (dotted lines) obtained by the GENFIT analysis of the SAXS data of N-His-aIF5A with 60 mM KCl. The protein concentration of each sample is: 0.5 mg/mL (blue); 1 mg/mL (green); 2 mg/mL (yellow); 5 mg/mL (orange); 10 mg/mL (red).

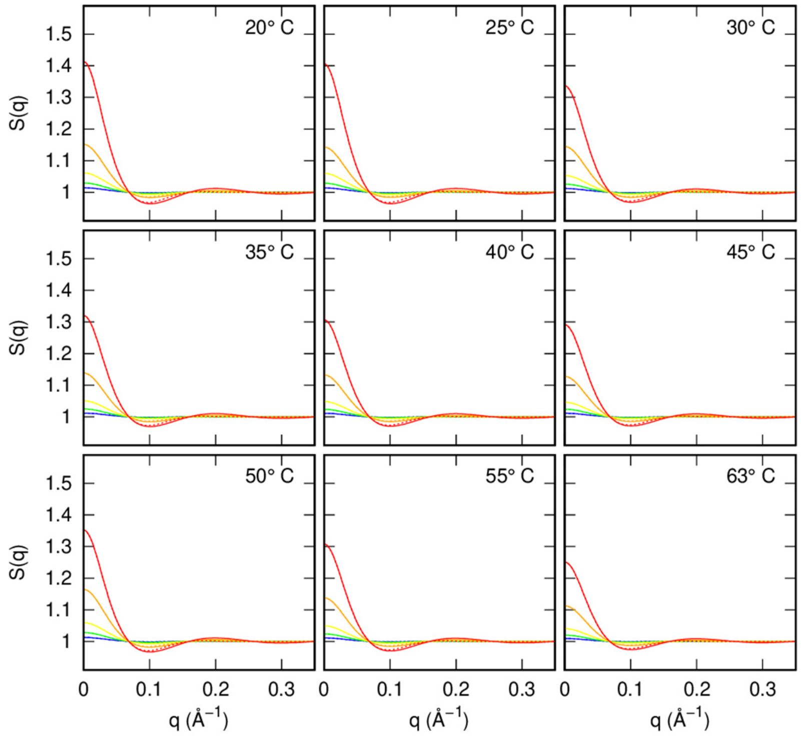

Figure S8. Structure factors $S(q)$ (solid lines) and "measured" structure factors $S_M(q)$ (dotted lines) obtained by the GENFIT analysis of the SAXS data of N-His-aIF5A with 1000 mM KCl. The protein concentration of each sample is: 0.5 mg/mL (blue); 1 mg/mL (green); 2 mg/mL (yellow); 5 mg/mL (orange); 10 mg/mL (red).

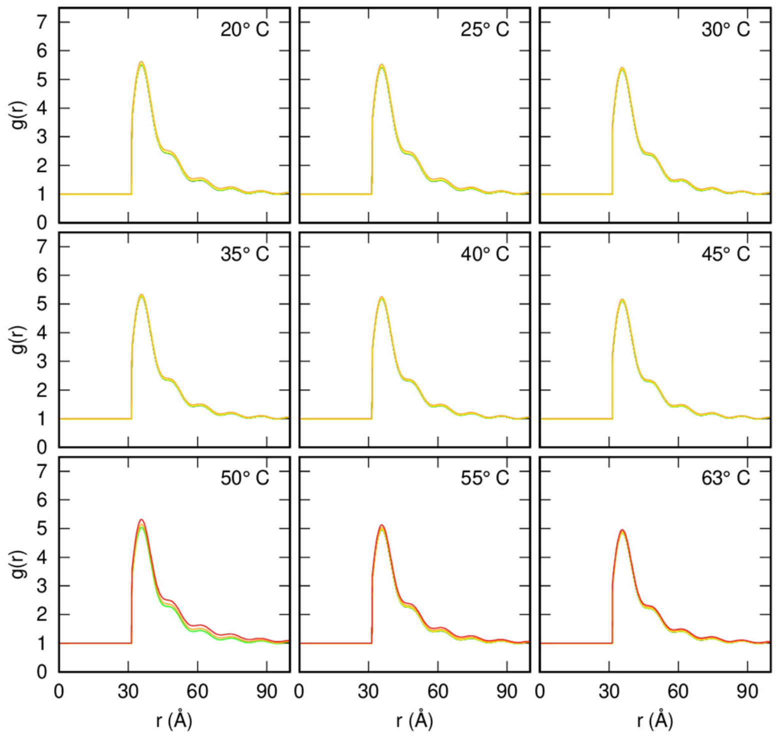

Figure S9. Radial correlation functions $g(r)$ calculated from the structure factors $S(q)$ obtained by the GENFIT analysis of the SAXS data of N-His-aIF5A with 60 mM KCl. The protein concentration of each sample is: 0.5 mg/mL (blue); 1 mg/mL (green); 2 mg/mL (yellow); 5 mg/mL (orange); 10 mg/mL (red).

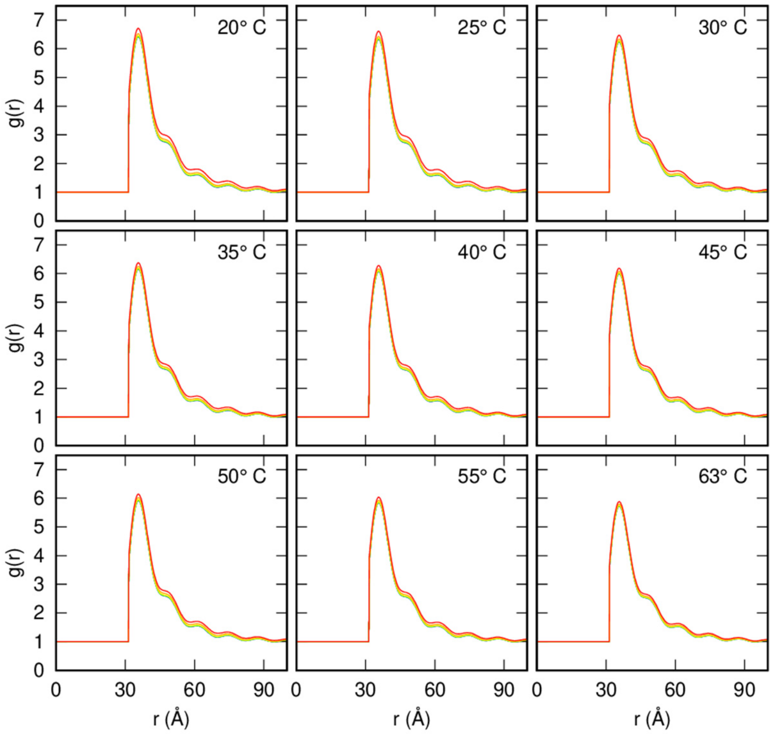

Figure S10. Radial correlation functions $g(r)$ calculated from the structure factors $S(q)$ obtained by the GENFIT analysis of the SAXS data of N-His-aIF5A with 1000 mM KCl. The protein concentration of each sample is: 0.5 mg/mL (blue); 1 mg/mL (green); 2 mg/mL (yellow); 5 mg/mL (orange); 10 mg/mL (red).

```
Hsa aIF5A     1 MAKEQKEVRDLQEGNYVMMEDAACQINAYSTAKPGKHGSAKARIEAEGVF     50
                |:.....|.:|:.|:||:::...|::...:.||.||||||||.:.|.|||
Sso aIF5A     1 MSITYTTVGELKVGSYVVIDGEPCRVVEVTKAKTGKHGSAKANVVAIGVF     50

Hsa aIF5A    51 DGKKRSLSQPVDAKIWVPIVNRKQGQIVSKESDTVAQVMDLETYETVTMQ    100
                .|.|::|..|||.::.|||:.:..|||::...:.: ||||||:|||..::
Sso aIF5A    51 SGAKKTLMAPVDQQVEVPIIEKHIGQIIADMGNKI-QVMDLESYETFEIE     99

Hsa aIF5A   101 IPGELD----IQADENIEYLEFEGQRKILQE-    127
                .|.|.:    |:.:..:||.|..|:|||::.
Sso aIF5A   100 KPTEDELASKIKPNAELEYWEIMGRRKIVRVK    131
```

Figure S11. Pairwise alignment of the amino acid sequences of *S. solfataricus* IF5A and *H. salinarum* IF5A. Wagner *et al.* (2007) detected residues are indicated in red. Hsa, *Halobacterium salinarum*; Sso, *Sulfolobus solfataricus*. *Halobacterium salinarum* is the other name for *Halobacterium sp.* NRC-1.

```
IF5A Saccharolobus solfataricus     1 MSITYTTVGELKVGSYVVIDGEPCRVVEVTKAKTGKHGSAKANVVAIGVFSGAKKTL
IF5A Sulfolobus islandicus          1 MSITYTTVGELKVGSYVVIDGEPCRVVEVTKAKTGKHGSAKANVVAIGVFSGAKKTL
IF5A Saccharolobus caldissimus      1 MGITYTTVGDLKVGSYVIIDGEPCRVVEITKAKTGKHGSAKANVVAIGVFSGAKKTL
IF5A Sulfolobus acidocaldarius      1 MSIQYTTVGDLKVGSYVMIDGEPCRVVEITKAKTGKHGSAKANVVAIGLFTGQKRSL
IF5A Acidianus brierleyi            1 MGVNYTTVGELKEGNYIVIDGEPCRVVDIQKAKTGKHGAAKANVVAVSVFTGSKKTL
IF5A Metallosphaera hakonensis      1 MGINYSTVGEMKEGSYIVIDGEPCRVVEVTKAKTGKHGSAKANIVAVSIFTGAKKTL
IF5A Sulfolobales archaeon          1 MGIQYATVGDLKVGSYVVIDGEPCRVVEITKAKTGKHGSAKANVVAIGLFTGAKKTL
IF5A Hyperthermus butylicus         1 MSVTYATLGELKVGSYIVIDGEPCRIVEMSKAKTGKHGSAKAHVVAVCLFSGNKKTL
IF5A Pyrolobus fumarii              1 MSYTYATVGDLKVGSYVIIDGEPCRIVEISKAKTGKHGSAKAHIVAIGLFTGSKKTL
IF5A Staphylothermus marinus        1 MSKTYATLGELKPGNFIIIDGEPCRIVEMSKAKTGKHGSAKAHVVAIGLFTGNKKTL
IF5A Staphylothermus hellenicus     1 MSKTYATLGELKSGNFIIIDGEPCRIVEMSKAKTGKHGSAKAHVVAIGLFTGNKKTL
IF5A Crenarchaeota archaeon         1 MSKTYATLGELKPGNFIVINGEPCRIVEMSKAKTGKHGSAKAHVVAIGLFTGNKRTL
IF5A Pyrobaculum calidifontis       1 MSTKYVEVGELKEGSYVVIDGEPCRVVEIEKSKTGKHGSAKARVVAVGVFDGAKRTL
IF5A Pyrobaculum aerophilum         1 MSTKYVEAGELKEGSYVVIDGEPCRVVEIEKSKTGKHGSAKARIVAVGVFDGGKRTL
IF5A Vulcanisaeta souniana          1 MSTKPTEAGSVKEGSYLMIDGEPCKIVEVEKSKTGKHGSAKVRIVGIGVFDNVKRTL
IF5A Thermoproteus uzoniensis       1 MSTKYVEVGELKEGSYIVIDGEPCRVVEIEKSKTGKHGSAKARVVAVGLFDNVKRTL
IF5A Thermoprotei archaeon          1 MS-KPVDAGSLKVGSFIVIDNEPCKIMEIEKSKPGKHGSAKCRIVAIGLFDGNKRSI
IF5A Thermoplasmata archaeon        1 MWTD-VEVRSLKPNRYIVIDDEPCKIVEITTSKPGKHGEAKARIVAIGVFDGQKRTV
IF5A Methanothermus fervidus        1 MAKKVVEVRTLKKGKYVVIDGEPSKIVGVTTSSPGKHGSAKMRIEAIGIFDGQKRSI
IF5A Infirmifilum lucidum           1 MSTRPEEAGNIKVGSFIVIDGEPCKVVEVEKSKTGKHGSAKARIVGIGFFDGAKRSI

IF5A Saccharolobus solfataricus    58 MAPVDQQVEVPIIEKHIGQIIADMGNKIQVMDLESYETFEIEKP-TEDELASKIKPN
IF5A Sulfolobus islandicus         58 MAPVDQQVEVPIIEKHIGQIIADMDDKIQVMDLETYETFEIEKP-TEDELASKIRPN
IF5A Saccharolobus caldissimus     58 MAPVDQQVEVPIIEKHVGQIIADMGDKIQVMDLETYETFEMEKP-TEDELVSKIRPN
IF5A Sulfolobus acidocaldarius     58 MAPVDQQVEVPIIEKHVGQILADKGDNLTIMDLESYETFDLEKP-TENEIVSKIRPG
IF5A Acidianus brierleyi           58 MAPVDQQVEVPIIEKHVGQILADTGEKLQVMDLNTYETFEMEKP-TEADLASKIRPG
IF5A Metallosphaera hakonensis     58 MAPVDSSVEVPIIEKHVGQVISNVGNKVQIMDLDTYETFDIDMP-TEEEVASKIRKD
IF5A Sulfolobales archaeon         58 MAPVDSQVEIPIIEKKVGQVLKVMGDKLQVMDLNTYETFEMEMP-KEPEIASKLADG
IF5A Hyperthermus butylicus        58 TAPVDARVEVPIIDKRIGQVIADMGDMVQIMDMETYETFEVEKP-KDEDLKSKLQPG
IF5A Pyrolobus fumarii             58 IAPVDQRVEVPIIEKRVGQVLAVTGDTVQIMDLETFDTFEAEKP-KDPKLAEQLQPG
IF5A Staphylothermus marinus       58 VAPVDQRVEVPVIEKRVGQIIADMGDLLQVMDMETFETFEVEKP-SDEKLREKLKPG
IF5A Staphylothermus hellenicus    58 VAPVDQRVEVPVIEKRVGQIIADMGDLLQVMDMETFETFEVEKP-SDEKLREKLQPG
IF5A Crenarchaeota archaeon        58 VAPVDQRVEVPIIEKRVGQVIADMGDMVQIMDMETFDTFEVEKP-EDEKLREKLQPG
IF5A Pyrobaculum calidifontis      58 SLPVDAQIEVPIIEKFTAQVLSISGDVIQLMDMRDYKTIEVPMKYVEEEAKGRLAPG
IF5A Pyrobaculum aerophilum        58 SLPVDAQVEVPIIEKFTAQILSVSGDVIQLMDMRDYKTIEVPMKYVEEEAKGRLAPG
IF5A Vulcanisaeta souniana         58 IVPADAQVEVPIIEKFVAQVVAKVGDSWQLMDLRNYTTFEVPQNQIEGDLGEKIEPG
IF5A Thermoproteus uzoniensis      58 SVPVDTQVEVPIIEKFTAQVLAISGDTVQLMDMRDYKTLEVPMKYVEEEAKGKLASG
IF5A Thermoprotei archaeon         57 VVPADSKVEVPIVNKKTAQVLALLQDTIQLMDLSTYEVYEVPVP-EDEDLRKRISPG
IF5A Thermoplasmata archaeon       57 VYPVKHKVRSPIIDKRQAQVLAVMGDEVQLMDLENYETFELPIP---EELRDQLEPG
IF5A Methanothermus fervidus       58 VKPVDSKIEVPVINKKVGQVLAIMGDTVQLMDLETYDTFEVPIP---EELKDKLTEG
IF5A Infirmifilum lucidum          58 VVPTDAKVDVPVIRKFNAQVVSMSGGYLQLMSLEDYNTFEVPMP-AEEEIKNKLTEG

IF5A Saccharolobus solfataricus   114 AELEYWEIMGRRKIVRVK
IF5A Sulfolobus islandicus        114 AELEYWEIMGRRKIVRVK
IF5A Saccharolobus caldissimus    114 AEVEYWEIMGRRKIVRVK
IF5A Sulfolobus acidocaldarius    114 AEIEYWSVMGRRKIVRVK
IF5A Acidianus brierleyi          114 AEIEYWTIMGRNKIVRVK
IF5A Metallosphaera hakonensis    114 AEVEYWEVMGRKKIVRVK
IF5A Sulfolobales archaeon        114 VEIEYWEIMGRKKIMRVK--EGV
IF5A Hyperthermus butylicus       114 VEVEYWVVMGRYMITRVRGAPKS
IF5A Pyrolobus fumarii            114 AEIEYWNVMGKRLIVRVR-PPRG
IF5A Staphylothermus marinus      114 VEVEYWVVMGKRMIIRTR
IF5A Staphylothermus hellenicus   114 VEVEYWVVMGKRMIIRTR
IF5A Crenarchaeota archaeon       114 VEVEYWIVMGKRMIVRTR
IF5A Pyrobaculum calidifontis     115 AEVEVWQILDRFKIVRVK
IF5A Pyrobaculum aerophilum       115 AEVEVWQILDRYKIIRVK
IF5A Vulcanisaeta souniana        115 IEVEVWDIAGRRKIVRVR
IF5A Thermoproteus uzoniensis     115 VEVEVWQILDRYKITRVK
IF5A Thermoprotei archaeon        113 VEVEVWEVLGRRKIMRIK
IF5A Thermoplasmata archaeon      111 KEVEYWEAMGKRKIMRVL----S
IF5A Methanothermus fervidus      112 AEVEYLEAMGKTKLLRVK
IF5A Infirmifilum lucidum         114 VEVEVWEVMGRYKIMRVR----S
```

Figure S12. Twenty Archaea IF5A sequences were aligned using COBALT MSA tool. Residues found using pairwise matching with *Halobacterium sp.* NRC-1 and previously discovered residues are represented in red and blue, respectively. *Saccharolobus solfataricus* is the other name for *Sulfolobus solfataricus*.

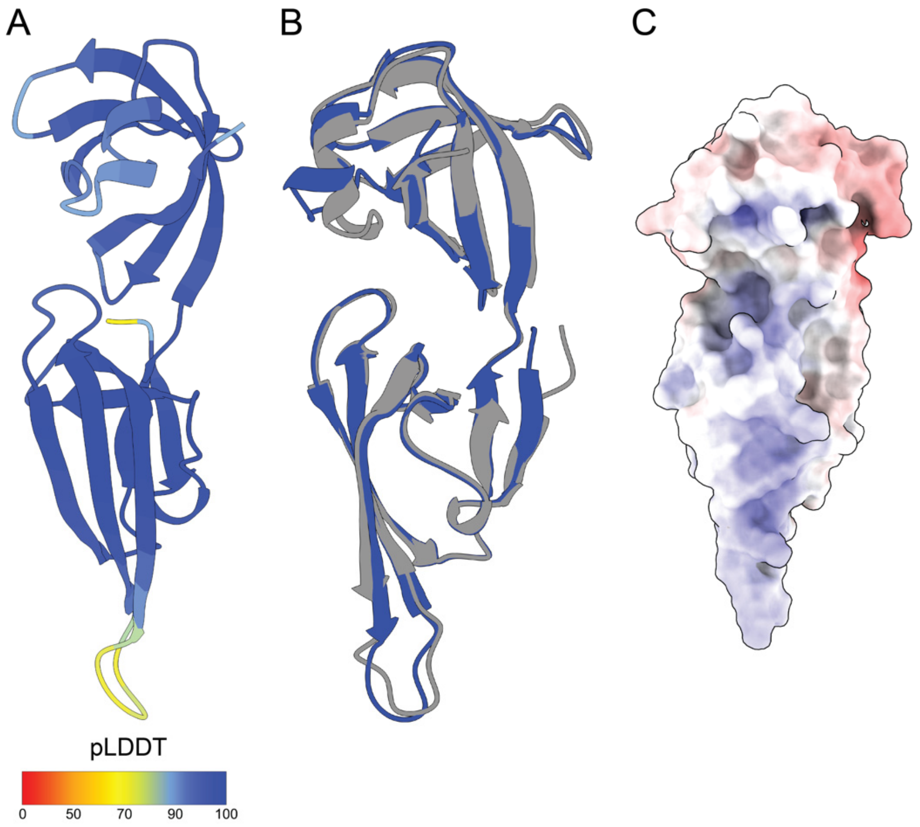

Figure S13. AlphaFold structural prediction for *S. solfataricus* aIF5A (i.e., AF-Sso-IF5A). (**a**) 3D ribbon representation of *S. solfataricus* aIF5A color coded using per-residue confidence metric (pLDDT). (**b**) Structure superimposition of AF-Sso-IF5A (blue) and SM-Sso-IF5A (grey). The calculated RMSD between the two structure was 0.843 Å. (**c**) AF-Sso-IF5A electrostatic potential surface (EPS): red is negative, blue is positive, and white is neutral. Surface potentials range between -10.0 kT/e (red) and 10 kT/e (blue). The EPS was prepared using the same procedure as in Figure 5C.

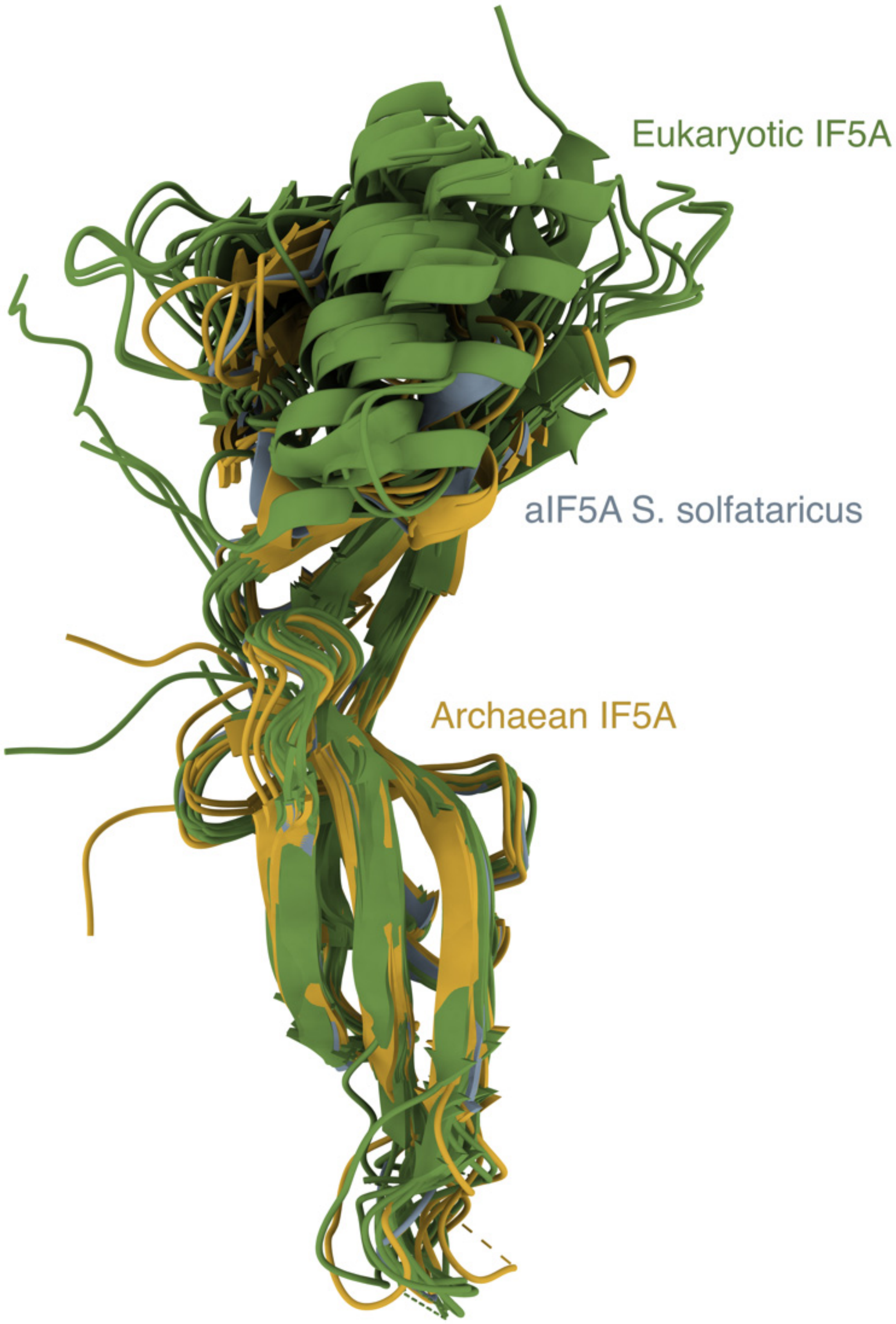

Figure S14. 3D ribbon Illustration of IF5A from Archaea and Eukarya species superimposed on the *S. solfataricus* aIF5A structure. *S. solfataricus* aIF5A, eukaryotic eIF5A, and archaeal aIF5A are represented in grey, green, and yellow, respectively.

**Oligonucleotide**	**Sequence (5´-3´)**
16S rRNA-FW	TTGGGATCGAGGGCTGAAAC
16S rRNA-RV	CTCACCCCTCTCCTACTCGG
2184-FW	GAGCAATTATATATCTTGACGAGGTTGACACG
2184-RV	AGCATACTTCGAAAGAATAAATTTCAGTTGC
0910-FW	CCGTTGCTAAAATATTAGGCGATTTGAAG
0910-RV	CTGGTAATAATTCCCTAACTTTAACTTCAGCC
nc98-FW	TAATACGACTCACTATAGGGATCTTTGGTTTAGCATCTCTC
nc98-RV	GTTAGAGAAAACGGAGAGG
2508sh-FW	AGATAATACGACTCACTATAGATGATTGTAGGATTTGCCGGAAAACT
2508sh-RV	CTGAAGGTCCTCCGTATTGGACATGTTC

Table S1: Oligonucleotides used in this work

Archaea	Eukarya
Sulfolobus solfataricus (Model and AlphaFold prediction)	*Arabidopsis thaliana* (3HKS Chain A)
Halobacterium sp. NRC-1 (Model)	*Homo sapiens* (3CPF Chain A)
Methanocaldococcus jannaschii (1EIF Chain A)	*Homo sapiens* (5DLQ Chain E)
Methanocaldococcus jannaschii (2EIF Chain A)	*Leishmania braziliensis* (1X6O Chain A)
Pyrobaculum aerophilum (1BKB Chain A)	*Leishmania Mexicana* (1XTD Chain A)
Pyrococcus horikoshii (1IZ6 Chain A)	*Naegleria fowleri* (7N4D Chain A)
	Saccharomyces cerevisiae (3ER0 Chain A)
	Saccharomyces cerevisiae (5DAT Chain F)
	Saccharomyces cerevisiae (5DC3 Chain F)
	Saccharomyces cerevisiae (6Q84 Chain C)
	Saccharomyces cerevisiae (6TNU Chain eI)
	Spodoptera frugiperda (5HY6 Chain A)

Table S2. Starting IF5A structures for computational analysis. When no PDB code is given, the structure is a model.

Supplementary References

1. O. Glatter; Kratky, O. Small Angle X-Ray Scattering. *Acta Crystallogr. Sect. A* **1983**, *39*, 500, doi:10.1107/S0108767383000926.
2. Spinozzi, F.; Ortore, M.G.; Nava, G.; Bomboi, F.; Carducci, F.; Amenitsch, H.; Bellini, T.; Sciortino, F.; Mariani, P. Gelling without Structuring: A SAXS Study of the Interactions among DNA Nanostars. *Langmuir* **2020**, *36*, 10387–10396, doi:10.1021/acs.langmuir.0c01520.
3. Frauenfelder, H.; Hartmann, H.; Karplus, M.; Kuntz, I.D.J.; Kuriyan, J.; Parak, F.; Petsko, G.A.; Ringe, D.; Tilton, R.F.J.; Connolly, M.L. Thermal Expansion of a Protein. *Biochemistry* **1987**, *26*, 254–261, doi:10.1021/bi00375a035.
4. Tarek, M.; Tobias, D.J. The Dynamics of Protein Hydration Water: A Quantitative Comparison of Molecular Dynamics Simulations and Neutron-Scattering Experiments. *Biophys. J.* **2000**, *79*, 3244–3257, doi:10.1016/S0006-3495(00)76557-X.
5. Spinozzi, F.; Beltramini, M. QUAFIT: A Novel Method for the Quaternary Structure Determination from Small-Angle Scattering Data. *Biophys. J.* **2012**, *103*, 511–521, doi:10.1016/j.bpj.2012.06.037.
6. Guinier, A.; Fournet., G. Small Angle Scattering of X-Rays. *J. Polym. Sci.* **1956**, *19*, 594, doi:https://doi.org/10.1002/pol.1956.120199326.
7. Malmberg, C.G.; Maryott, A.A. Dielectric Constant of Water from 0 0 to 100 0 C. In Proceedings of the Physic; 2011.
8. Bassani, F.; Zink, I.A.; Pribasnig, T.; Wolfinger, M.T.; Romagnoli, A.; Resch, A.; Schleper, C.; Bläsi, U.; La Teana, A. Indications for a Moonlighting Function of Translation Factor AIF5A in the Crenarchaeum Sulfolobus Solfataricus. *RNA Biol.* **2019**, *16*, 675–685, doi:10.1080/15476286.2019.1582953.
9. Bassani, F.; Romagnoli, A.; Cacciamani, T.; Amici, A.; Benelli, D.; Londei, P.; Märtens, B.; Bläsi, U.; La Teana, A. Modification of Translation Factor AIF5A from Sulfolobus Solfataricus. *Extremophiles* **2018**, *22*, 769–780, doi:10.1007/s00792-018-1037-4.
10. Yao, M.; Ohsawa, A.; Kikukawa, S.; Tanaka, I.; Kimura, M. Crystal Structure of Hyperthermophilic Archaeal Initiation Factor 5A: A Homologue of Eukaryotic Initiation Factor 5A (EIF-5A). *J. Biochem.* **2003**, *133*, 75–81, doi:10.1093/jb/mvg011.
